# Supplementary material for: Reconciling Mining with the Conservation of Cave Biodiversity: A Quantitative Baseline to Help Establish Conservation Priorities
Source: PLoS One. 2016 Dec 20;11(12):e0168348. doi: 10.1371/journal.pone.0168348 (PMC5173368; doi:10.1371/journal.pone.0168348)
Supplement: S1 Dataset — (ZIP) [file pone.0168348.s002.zip › Taxa/Serra Norte/SN_2013/N4E/Lista N4E-15.pdf]

CAVIDADE N4E-0015

Classe	Ordem	Fam/Outros	Gên/Outros	Espécie	S-U	U-S	Zon.
Annelida	Oligochaeta			jovem		5	P
Arachnida	Acari	(Parasitengonina)		sp.2		X	P
Arachnida	Acari	(Uropodina)		jovem		X	P
Arachnida	Acari	Argasidae	*Ornithodoros*	sp.1	X	X	P
Arachnida	Acari	Ixodidae	Amblyomma	sp.1	X		
Arachnida	Acari	Laelapidae		sp.3		X	P
Arachnida	Acari	Mesostigmata		sp.3		X	P
Arachnida	Amblypygi	Charinidae	*Charinus*	sp.1		3	P
Arachnida	Amblypygi	Phrynidae	*Heterophrynus*	*longicornis*	X		
Arachnida	Araneae	Caponiidae		sp.1	X	X	P
Arachnida	Araneae	Corinnidae		jovem	1		
Arachnida	Araneae	Ctenidae		jovem		1	P
Arachnida	Araneae	Filistatidae		sp.1	X	X	P
Arachnida	Araneae	Ochyroceratidae	*Ochyrocera*	sp.	X		
Arachnida	Araneae	Ochyroceratidae	*Ochyrocera*	sp.1		X	P
Arachnida	Araneae	Ochyroceratidae	*Ochyrocera*	sp.3		X	P
Arachnida	Araneae	Ochyroceratidae	*Speocera*	sp.1		X	P
Arachnida	Araneae	Oonopidae	aff. *Triaeris*	sp.2		X	P
Arachnida	Araneae	Oonopidae	aff. *Xyccarph*	sp.1		X	P
Arachnida	Araneae	Pholcidae	*Mesabolivar*	sp.1	X	X	P
Arachnida	Araneae	Scytodidae	*Scytodes*	sp.	X	1	P
Arachnida	Araneae	Segestriidae		jovem	X		
Arachnida	Araneae	Theridiosomatidae	*Plato*	sp.1	X	X	P
Arachnida	Opiliones	Escadabiidae		sp.1	X	X	P
Arachnida	Opiliones	Stygnidae	*Protimesius*	*gracilis*	X		
Arachnida	Palpigradi	Eukoeneniidae	*Allokoenenia*	sp.1		X	P
Arachnida	Pseudoscorpiones	Cheliferoidea		sp.1		X	P
Arachnida	Pseudoscorpiones	Chernetidae		sp.2		X	P
Arachnida	Pseudoscorpiones	Chthoniidae	*Pseudochthonius*	sp.1		X	P
Arachnida	Pseudoscorpiones	Chthoniidae	*Pseudochthonius*	sp.3		X	P
Chilopoda	Scolopendromorpha	Scolopocryptopidae	*Tidops*	jovem		1	P
Chilopoda	Scutigeromorpha	Pselliodidae	*Sphendononema*	*guildingii*	X		
Diplopoda	Polydesmida	Chelodesmidae		sp.	X		
Diplopoda	Polydesmida	Pyrgodesmidae		sp.1	X	X	P
Diplopoda	Siphonophorida	Siphonophoridae		jovem		1	P
Diplopoda	Spirostreptida	Pseudonannolenidae	*Pseudonannolene*	sp.1	X		
Entognatha	Collembola	Cyphoderidae		sp.1		X	P
Entognatha	Collembola	Isotomidae		sp.1		X	P
Entognatha	Collembola	Paronellidae		sp.1		X	P
Entognatha	Collembola			sp.	X		
Entognatha	Diplura	Campodeidae		sp.1	X	X	P
Insecta	Blattodea			jovem	X	2	P
Insecta	Coleoptera	Carabidae		sp.3		X	P
Insecta	Coleoptera			jovem	X	X	P
Insecta	Diptera	Cecidomyiidae	Cecidomyiinae	sp.	X	X	P
Insecta	Diptera	Ceratopogonidae		sp.		X	P
Insecta	Diptera	Culicidae	*Culex*	sp.		X	P
Insecta	Diptera	Psychodidae	*Sciopemyia*	*sordellii*	X	X	
Insecta	Diptera	Tipulidae	Tipulinae	sp.		X	P
Insecta	Heteroptera	Cydnidae		jovem		X	P
Insecta	Heteroptera	Reduviidae	*Vescia*	sp.	X		
Insecta	Heteroptera	Reduviidae	*Zelurus*	sp.	X		
Insecta	Heteroptera	Tingidae		jovem		X	P
Insecta	Homoptera	Cixiidae		jovem	X	X	P
Insecta	Hymenoptera	Bethylidae		sp.	X		

Insecta	Hymenoptera	Formicidae	*Camponotus*	*atriceps*	X		
Insecta	Hymenoptera	Formicidae	*Camponotus*	sp.1		X	P
Insecta	Hymenoptera	Formicidae	*Gnamptogenys*	sp.1		X	P
Insecta	Hymenoptera	Formicidae	*Pachycondyla*	*arhuaca*	X		
Insecta	Hymenoptera	Formicidae	*Pachycondyla*	sp.2		X	P
Insecta	Hymenoptera	Formicidae	*Pheidole*	sp.2		X	P
Insecta	Hymenoptera	Formicidae	*Solenopsis*	sp.1		X	P
Insecta	Isoptera	Termitidae	*Labiotermes*	sp.		X	P
Insecta	Isoptera	Termitidae	*Nasutitermes*	sp.	X	X	P
Insecta	Lepidoptera	Tineoidea		sp.	X	X	
Insecta	Neuroptera	Myrmeleontidae		jovem		X	P
Insecta	Orthoptera	Gryllidae		sp.		1	P
Insecta	Orthoptera	Phalangopsidae	*Phalangopsis*	sp.	X	2	P
Insecta	Plecoptera			jovem		1	P
Insecta	Psocoptera	Psyllipsocidae		jovem		X	P
Gastropoda	Pulmonata	Systrophiidae	*Happia*	sp.		X	P
Malacostraca	Isopoda	Armadillidae		sp.1		X	P
Malacostraca	Isopoda	Scleropactidae	*Circoniscus*	*buckupi*	X	X	P
Nematoda				sp.1		X	P
Amphibia	Anura			sp.	3	2	P
Mammalia	Chiroptera	Emballonuridae	*Peropteryx*	sp.	10		P
Mammalia	Chiroptera	Phyllostomidae	*Carollia*	*perspicillata*		1	P
Mammalia	Rodentia	Cricetidae	*Rhipidomys*	sp.		3	P
Reptilia	Squamata	Gekkonidae	*Thecadactylus*	sp.		1	P
